# Implementation of the Anchor-Based Indirect Comparison Method for Equivalence Margin Derivation in Biosimilar Development

**DOI:** 10.3390/ph18030285

**Published:** 2025-02-20

**Authors:** Claudia Hemmelmann, Rachid El Galta, Jessie Wang, Susanne Schmitt, Ramin Arani

**Affiliations:** 1 Hexal AG, Industriestr. 25, D-83607 Holzkirchen, Germany; 2 Sandoz Inc., 100 College Road West, Princeton, NJ 08540, USA

**Keywords:** biosimilarity, equivalence margin, indirect approach, network meta-analysis

## Abstract

**Background/Objectives**: To derive the equivalence margin (EQM), typically, a “classical” meta-analysis on direct within-trial estimation of the effect size of the reference drug compared to the placebo or standard of care is performed: a certain factor of the 95% confidence interval for the pooled treatment effect compared to placebo is used. However, treatment regimens in many indications are becoming more complex (e.g., combination treatments), and for most of these clinical study data, direct comparisons are not available. On the other hand, data for the comparison of the common treatment to the reference treatment in one study and to the placebo in another study are available in some situations. **Methods**: In such situations, an anchor-based indirect comparison can be applied to estimate the treatment effect of Reference vs. Placebo. This treatment effect (Reference vs. Placebo) can be estimated by calculating the difference of the two treatment effects and the variance as the sum of both variances. The 95% confidence interval of this estimated treatment effect can then be used to derive the EQM. To alleviate any concerns about the underlying assumptions of transitivity and consistency, multiple sensitivity analyses can be performed. **Results**: We present a case study for deriving the EQM using the anchor-based indirect comparison along with sensitivity analyses (i.e., direct comparison against similar reference drug, the impact of variation of treatment effect on Comparator, and effect size Reference vs. Placebo, including trial data with slightly different population characteristics) for a planned efficacy trial in the biosimilar setting. **Conclusions**: An anchor-based indirect comparison for EQM derivation is an approach health authorities can agree to if sufficiently supported through other means, e.g., relevant sensitivity analyses.

## 1. Introduction

Biosimilar development refers to the process of creating a biologic drug that is similar to an existing approved biologic drug, also known as a reference drug. Biosimilars are designed to be highly similar to the reference drug in terms of quality, safety, and efficacy but are not identical due to the complex nature of biologic drugs and the inherent variability in their manufacturing process.

The biosimilarity is typically established in a stepwise process assessing similarity criteria agreed upon with health authorities with respect to quality, analytical, pharmacokinetics, efficacy, and safety parameters. The biosimilar developer must demonstrate that the biosimilar has the same mechanism of action, pharmacokinetics, and clinical effect as the reference drug (Section 351(i) of the Public Health Service Act).

Biosimilar clinical development is a lengthy process that requires identifying the most sensitive target population and the most sensitive pharmacokinetics/clinical endpoints, which will be able to demonstrate potential residual differences between biosimilar and reference drugs. This is performed through an equivalence design biosimilar study for which it is necessary to determine the equivalence margin (EQM). The EQM represents a maximum clinically acceptable difference in effect size of the test drug compared to the reference drug.

**Figure 1:**
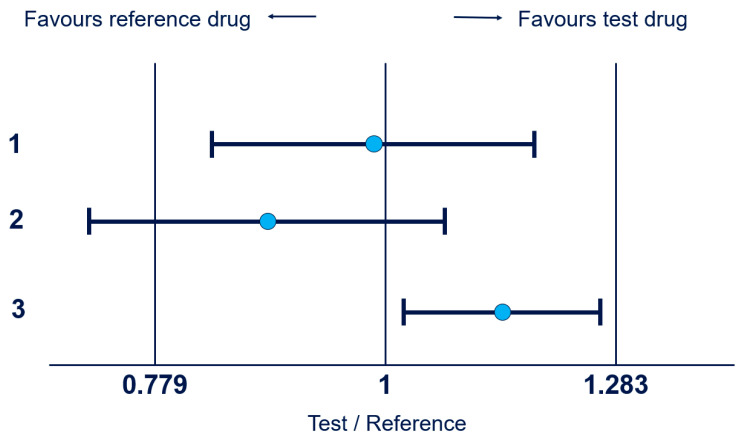
Examples of equivalence testing with CIs (blue circles indicates the point estimate).

For example, to confirm no clinically meaningful differences in effectiveness, typically, a phase 3 clinical study with an equivalence design is performed based on the guidance provided by health authorities [1,2]. To show biosimilarity, two one-sided tests are usually conducted on the primary efficacy endpoints. Similarity is concluded when a two-sided confidence interval (CI) for the estimate of treatment comparison is fully contained within a pre-specified margin (see Figure 1). The approach depicted in this paper can be applied to various estimates of treatment difference, such as a difference in means for continuous outcomes and a risk difference or log risk ratio for binary outcomes.

1. Equivalence met: CI contained entirely within the margin of (0.779, 1.283).
2. Equivalence not met: CI partially outside the margin of (0.779, 1.283).
3. Equivalence met, but an additional explanation is needed for why the CI does not contain the equality point of 1.

The EQM can be established through (a) scientific experts’ input; (b) health authorities’ precedence or widely accepted agreement for a given indication, such as EQM of (0.8, 1.25) for PK/PD parameters; or (c) estimated treatment effects from agreed comparable historical clinical studies [3,4,5].

The classical approach for estimating the reference drug treatment effect in order to derive the equivalence margin (EQM) involves performing a meta-analysis of randomized clinical superiority trials where the treatment arms of interest (reference product and placebo) are directly compared. This is achieved by taking a fraction of the lower limit of the 95% CI of the pooled effect, ensuring that a certain factor of the treatment effect of the reference product compared to placebo is maintained [3,4]. However, as treatment regimens in various indications rapidly evolve and become more complex, historical trials that directly compare the reference product to placebo may not be available. For example, this is the case when the standard of care changes or when treatments are combined.

In such situations an indirect approach [6] can be applied to evaluate the relative effectiveness between two treatments of interests. Indirect comparison methods are a special case of network meta-analysis [7]. They synthesize evidence from separate trials that involve a common comparator arm when direct comparisons between the treatments of interest are not available. This approach allows for the estimation of relative treatment effects using the common comparator as a bridge. A thorough description and best practices for conducting indirect comparison can be found elsewhere, e.g., [8,9,10].

In this paper, following the approach outlined by Harrer et al. [11], we provide a case example of how we estimated the treatment effect and derived a margin from an anchor-based indirect comparison approach in a biosimilar efficacy study where relevant historical data were not available within one trial, but across two trials. We estimated the point estimate for the treatment effect across trials by calculating the difference of the two treatment effects. The variance was estimated as the sum of both variances from each trial. We derived the margin using the lower bound of the 95% CI of this estimated treatment effect. To help alleviate the concern of potential disparity of a treatment comparison based on direct evidence versus indirect evidence, we propose three different sensitivity analyses. This approach for indirect margin derivation was successfully applied and agreed upon with health authorities.

The paper is organized as follows. In Section 2, we showcase the method applied to a case study including their interpretations. A discussion of the results in the context of their assumptions is given in Section 3. Details of the applied methodology are presented in Section 4.

## 2. Results

To illustrate the anchor-based indirect margin derivation approach and the performed sensitivity analyses, we present a case study for a planned efficacy trial in the biosimilar setting. In our fictional case study, we use response rate as an endpoint (see Table 1).

### 2.1. Anchor-Based Indirect Margin Derivation

Given the response rate as an endpoint, we compare the two treatments in each trial (Anchor vs. Reference; Anchor vs. Placebo) directly with a treatment rate ratio.

For the indirect comparison of Reference vs. Placebo, the ratio is the ratio of the two ratios (of Trials 1 and 2), and the variance represented by SE(ln(ratio)) is the sum of the individual trial variances. The exact formulas are shown in Table 2.

The EQM based on different amounts of effect size maintained are displayed in Table 3.

For our case study, we will use a preservation factor of 60% and therefore derive an equivalence margin of (0.779, 1.283) using the anchor-based indirect margin derivation approach.

### 2.2. Sensitivity Analysis 1: Direct Margin Derivation for When Anchor Has Similar Treatment Effect as Reference Treatment

In the scenario where the anchor drug has a similar treatment effect as the reference treatment (Anchor ≈ Reference), we can support the indirect margin derivation of <Reference vs. Placebo> with a direct within-trial derived margin of <Anchor vs. Placebo>. The derivation of the margin using the <Anchor vs. Placebo> treatment effect has its own drawback by replacing Reference with Anchor and thereby increasing the uncertainty with the estimated margin for <Reference vs. Placebo>. However, the data come from the same trial reducing the consistency concern. If Reference can be shown to have a similar treatment effect as Anchor and the margin derived for <Anchor vs. Placebo> is not tighter than the one derived for <Reference vs. Placebo>, this should give some more assurance that the indirectly derived treatment effect is not very different from a directly derived treatment effect and thereby margin.

For a preservation factor of 60%, we derive an equivalence margin of (0.724, 1.381) for <Anchor vs. Placebo>. When comparing this EQM with the proposed EQM based on the anchor-based indirect comparison for <Reference vs. Placebo>, it can be seen that it is wider for maintaining 60% of the treatment effect size. The results of the sensitivity analysis support the proposed equivalence margin of (0.779, 1.283).

### 2.3. Sensitivity Analysis 2: Impact of Variation of Treatment Effect on Reference and Effect Size Reference vs. Anchor

To provide further assurance on the robustness of the indirect margin derived for <Reference vs. Placebo>, we vary the treatment effect of both Reference as well as <Reference vs. Anchor> and confirm its impact on the derived margin.

This section gives an overview only. Details on formulas and methods can be found in Section 4.

#### 2.3.1. Impact of Variation of the Treatment Effect of Reference Treatment

For the impact of variation of the treatment effect of reference treatment, we vary the assumed treatment effect of Reference (in the range of its 95% CI of 0.356 and 0.494, see Table 1) while keeping the treatment effect of Anchor constant. For each value of Reference in this range, we then derive the preservation factor we would have for a margin of (0.779, 1.283) as derived with the indirect approach in Section 2.1.

**Figure 2:**
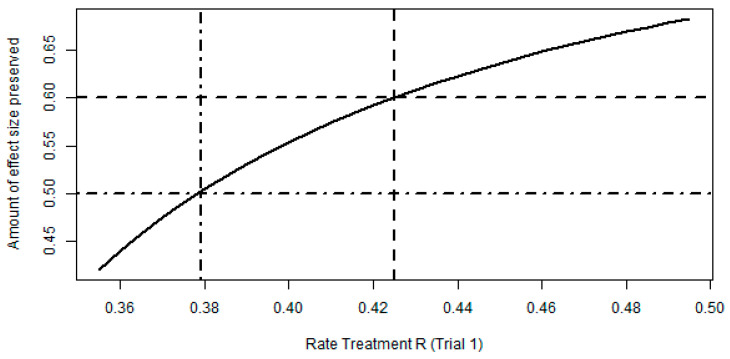
Impact of change in Rate Treatment Reference (R) on preservation factor.

As can be seen in Figure 2, when the rate of reference treatment is as low as 38.0%, the indirect comparison approach can still maintain a preservation factor of at least 0.5 with the proposed equivalence margin of (0.779, 1.283) (Figure 2, see dash-dotted line). The dashed line indicates the proposed preservation factor of 60% based on the observed treatment effects in Trial 1.

**Figure 3:**
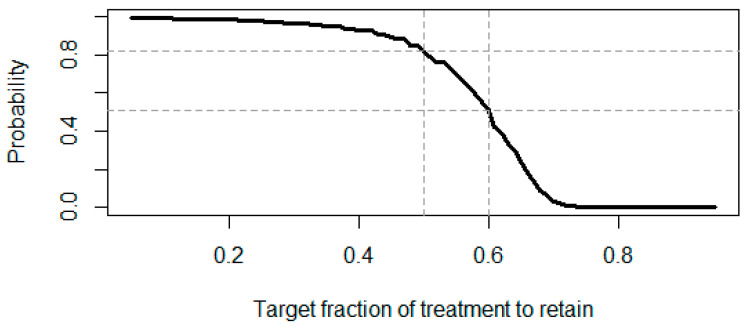
The probability of achieving at least the target preservation factor of treatment is retained based on the posterior distribution of response rate in active treatment.

One can even run simulations to understand the likelihood of each preservation factor (for R code, see Supporting Information). The simulation result for our case study is shown in Figure 3.

Assuming a targeted retained effect size of 60%, there is a probability of about 50.6% that we would achieve that target fraction within the trial. In case the targeted retained effect size is at least 50% in this sensitivity analysis, then the probability to achieve that target fraction is about 82.0%.

#### 2.3.2. Impact of Variation of Treatment Difference Reference vs. Anchor

For the impact of variation of the treatment effect of reference treatment, we vary the assumed treatment effect of Reference (in the range of its 95% CI of 0.356 and 0.494, see Table 1) while keeping the treatment effect of Anchor constant. For each value of Reference in this range, we then derive the preservation factor we would have for a margin of (0.779, 1.283) as derived with the indirect approach in Section 2.1.

For the impact of variation of treatment difference <Reference vs. Anchor>, we assumed a treatment difference of <Reference vs. Anchor> of 4.5% as observed, 6.0%, 7.5%, and 9.0% twice as observed and then repeated the below steps for each fixed treatment difference of Reference vs. Anchor.

1. Vary the effect of Reference between 0.356 and 0.494 (range of 95% CI).
2. Based on the given difference of <Reference vs. Anchor> and the effect of Reference, derive the value of Anchor.
3. For each value pair (Reference vs. Placebo, Reference) derive the preservation factor for the margin of (0.779, 1.283) as derived with the indirect approach in Section 2.1.

**Figure 4:**
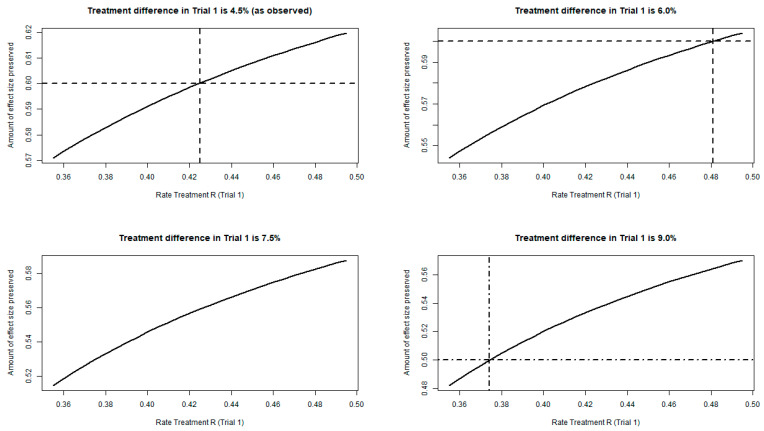
Impact of change in response rate of treatment difference Reference vs. Placebo on preservation factor (Note: The dash-dotted line represents the threshold of 50% maintenance of the treatment effect and the dashed line the threshold of 60% maintenance.).

We then graphically displayed the effect of Reference versus preservation factor for each treatment difference <Reference vs. Anchor>; see Figure 4.

In case of a treatment difference of approximately 4.5%, the preservation factor is at least 57.1%; for a 6.0% treatment difference, at least 54.4%; for a 7.5% treatment difference, at least 51.4%; and for 9.0% treatment difference, the preservation factor is at least 48.2%.

**Figure 5:**
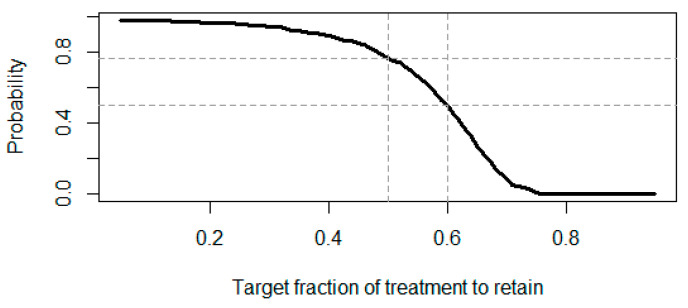
Probability of achieving at least the target preservation factor of treatment based on the posterior distribution of response rates in Treatment R and A.

One can even run simulations to understand the likelihood of each preservation factor. An R code is provided as Supporting Information for conducting this simulation, which also uses the Netmeta package [12,13] for performing the analysis of the data. The output is shown in Figure 5.

Assuming a targeted retained effect size of 60%, there is a probability of 50.2% that we would achieve that target fraction within the trial. For a targeted retained effect size of 50% there is even a probability of 76.7% that we would achieve the target fraction within the trial.

The presented sensitivity analyses demonstrated that the proposed equivalence margin of (0.779, 1.283) can maintain a preservation factor of around 0.5 with variation in rate values in nearly all possible cases.

### 2.4. Sensitivity Analysis 3: Including Trial Data with Difference in Population Characteristics

Assume further trial data are available from a trial with a slightly different population. We can use these data to understand the impact of this difference on our margin. This will test the robustness of our margin against deviations in population characteristics.

In our example, let it be further trial data for the <Anchor vs. Placebo> comparison (Table 4).

**Figure 6:**
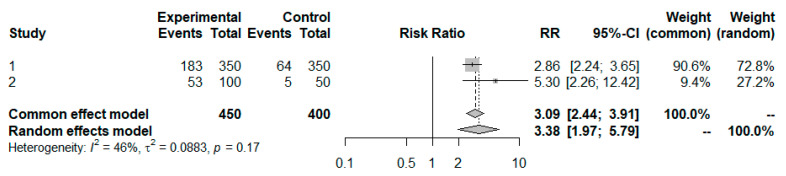
Forest plot of meta-analysis (Mantel–Haenszel method was used in the meta-analysis, created with R package meta [12]).

As the trial characteristics now differ, we can anticipate between-trial heterogeneity in the true effects. Using a random effects model (see details in Section 4), we obtain an overall estimate of the effect size of <Anchor vs. Placebo> as depicted In Figure 6 and as part of Table 5. Figure 6 displays forest plots of the treatment comparison in response rates from Trial 2 and Trial 3. The heterogeneity τ2 index indicates that there is some between-study variability. Nevertheless, it is appropriate to pool the trials based on a random-effect model to obtain an overall estimate of the effect size. In addition, the observed between-study variability supports the rationale for excluding Trial 3 from the anchor-based indirect comparison.

Using the same approach as described in Section 4, we derive a treatment effect of <Reference vs. Placebo> as depicted in Table 5.

For a preservation factor of 60%, we obtain an equivalence margin of (0.807, 1.240). By applying this approach, our proposed equivalence margin of (0.779, 1.283) maintained 53.6% of the treatment effect (using the lower limit of the 95% CI).

The inclusion of Trial 3 into the anchor-based indirect comparison, despite the highlighted differences of the enrolled populations, would still support our proposed equivalence margin.

## 3. Discussion

The primary objective of using an indirect estimation method in this case study is to derive the equivalence margin for the comparison of the proposed biosimilar with the reference product. In the absence of within-trial data to estimate the effect size of <Reference vs. Placebo>, and with only comparative data available for both Reference and Placebo against the same comparator in separate trials, an anchor-based approach can be used. This method uses indirect comparisons to estimate the effect size of <Reference vs. Placebo>. The 95-95 method [4], known for its conservative nature, was used to determine the margin as a fraction of the lower limit of 95% confidence interval of the effect size. The fraction is selected based on clinical justification of the resulting margin and ensures the preservation of at least 60% of the effect size, thereby ensuring indirect superiority and accounting for potential departure from the constancy assumption.

This method ensures that the derived equivalence margin is both robust and reliable. The variance was estimated as the sum of the variances from both comparisons. This conservative approach ensures that the uncertainty in the treatment effect estimate is adequately accounted for, providing a more conservative and reliable margin.

Network meta-analysis relies on several key assumptions, such as similarity, consistency, and homogeneity [10,14]. To ensure the comparability and validity of the indirect estimates, the included studies in the analysis should be similar in terms of patient populations, interventions, and outcomes. In this hypothetical case study, we assumed that the included studies were similar. Likewise, the similarity assumption was evaluated and confirmed in our real case study. However, due to confidentiality constraints, we cannot demonstrate this in the article. Nevertheless, it is a crucial point that needs to be addressed.

In general, treatment effects should be homogeneous across the included trials. Therefore, we carefully selected the included trials and excluded one trial from our main analysis based on a qualitative assessment of the trial characteristics. This was disputed by the Agency. Therefore, a sensitivity analysis shows how one can consider historical study data that meet some but not all the requirements in terms of population characteristics or treatment schedules to provide additional assurance on the robustness of the selected margin.

Direct and indirect evidence should be consistent, i.e., direct treatment comparisons and indirect comparisons should lead to the same conclusions. In the absence of direct comparisons, it is difficult to verify the consistency assumption. This limitation needs to be acknowledged as it affects the reliability of the results of the indirect comparisons. In this case study, we proposed two different sensitivity analyses to address this concern, which were accepted by health authorities. The first sensitivity analysis uses an available direct comparison of Anchor against Placebo. Of course, this is only meaningful if the Anchor is similar to the Reference, which is not often the case. This can already provide some assurance on the consistency assumption when the preserved effect size based on the comparison Anchor to Placebo is comparable to the preserved effect size based on indirect comparison Reference to Placebo. The second proposed sensitivity analysis ensures the robustness of the approach with different assumptions for the effect of reference and the effect size of <Reference vs. Anchor> in terms of how much effect would be retained for each scenario.

Deviations from similarity, consistency, and homogeneity assumptions can have a significant impact on the constancy assumption. To mitigate this risk, a preservation factor of 60% was selected. The sensitivity analyses help to assess the robustness of the results under various scenarios, ensuring that the derived equivalence margin remains valid even if some assumptions are violated. Our sensitivity analysis showed that the preservation factor is at least 50% by applying the equivalence margin.

The proposed indirect approach is both practical and pragmatic, and it may be the most feasible method for establishing a margin when direct head-to-head placebo-controlled trials of the reference treatment are unavailable.

However, it is important to acknowledge that the method has limitations inherent to network meta-analysis in particular and to standard meta-analysis in general, which can impact the results if not carefully considered. These limitations are briefly discussed above. For a detailed elaboration on the limitations of meta-analysis due to the poor quality of included studies, heterogeneity among studies, and potential publication bias, we refer to [15]. Additionally, Song et al. (2003) [16] investigated the validity of adjusted indirect comparisons using data from published meta-analyses of randomized trials, and Song et al. (2009) [17] discussed methodological issues regarding the basic assumptions of homogeneity, similarity, and consistency in the use of indirect comparisons. It is essential to carefully consider these limitations both prior to and during the conduct of indirect comparisons analysis, as well as when interpreting the results. To maintain the similarity of the studies included, rigorous selection criteria should be applied. When more than two studies are included, sensitivity analysis can be performed by excluding some studies to test the robustness of the findings. Homogeneity among studies can be inspected by performing subgroup analyses to ensure that patient characteristics and study designs are comparable. Statistical tools to formally assess the consistency assumption in network meta-analysis are available, but they require both direct and indirect comparisons, which allows for the evaluation of whether the direct evidence aligns with the indirect evidence or the existence of multiple pathways of indirect comparisons to check for consistency across these different pathways.

As a future work, the impact of population heterogeneity on the outcome and the consistency could be explored in more detail in a well-defined simulation study. For example, one could examine the effect of missing information (e.g., if direct comparisons within Trials 1 and 2 were available versus if these direct comparisons were not available).

While our primary focus has been on biosimilar development, the anchor-based indirect comparison method holds significant potential for broader applications beyond biosimilars. This methodology can also be applied to other kinds of endpoints and to margin justification for non-inferiority trials, where demonstrating that a new treatment is not significantly less effective than an established therapy is crucial. Furthermore, the approach can be applied in various therapeutic areas where direct comparative trials are not feasible. For example, in oncology, where numerous treatment regimens exist, and direct comparisons are not there, the anchor-based method can facilitate robust comparisons. By leveraging this approach in broader contexts, researchers can enhance the evaluation of interventions in diseases with diverse treatment landscapes, enabling more informed clinical and regulatory decisions across different medical fields.

## 4. Materials and Methods

The fixed margin approach, also known as the 95-95 method [4], was used to determine the equivalence margin as a fraction of the lower limit of 95% confidence interval of the effect size. This calculation typically uses meta-analytic methods with data from previous studies to obtain a 95% confidence interval around the estimated difference between the reference and placebo. In our case, there is no historical trial available that directly compared the reference treatment to placebo. Therefore, an anchor-based indirect comparison was applied, and this approach is described in detail in this section. In addition, the different sensitivity analyses are motivated and described.

### 4.1. Anchor-Based Indirect Margin Derivation

**Figure 7:**
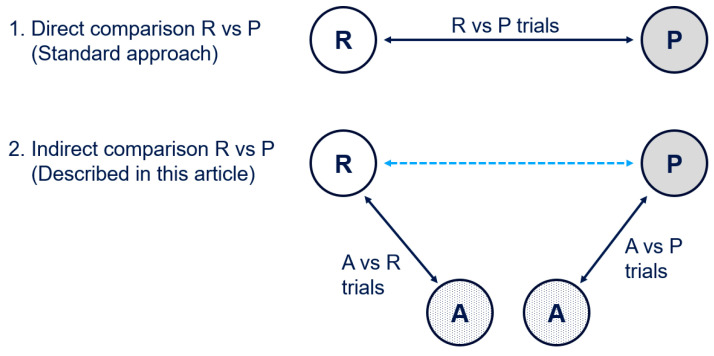
Direct comparison vs. anchor-based indirect comparison; R = Reference, A = Anchor, P = Placebo (blue dotted line indicated the indirect comparison).

Assume historical data comparing the Reference (R) to Placebo (P) is not available within one trial, but across two trials that both have a common comparator, the anchor (A), i.e., we are in the second situation of Figure 7. Of note, we refer to placebo in this article, but this does not exclude active background therapy, e.g., a comparison drug + background therapy versus background therapy would follow the same principles.

How do you calculate the margin of treatment effect <Reference vs. Placebo> in the second scenario?

The treatment effect (Reference (R) vs. Placebo (P)) can then be estimated by calculating the difference of the two treatment effects,

$${\hat{\theta }}_{R,P}^{indirect}={\hat{\theta }}_{A,R}^{direct}-{\hat{\theta }}_{A,P}^{direct}$$

and the variance as the sum of both variances [8]

$$Var\left({\hat{\theta }}_{R,P}^{indirect}\right)=Var\left({\hat{\theta }}_{R,A}^{direct}\right)+Var\left({\hat{\theta }}_{A,P}^{direct}\right).$$

Note that instead of the difference of the two treatment effects, the ratio of the treatment effects was used in our example in Section 2.

With the effect and the variance of the effect established, the margin can be derived as a proportion of the lower bound of the 95% CI for the effect size depending on the relevant preservation factor of effect size as for the classical margin derivation.

As described by Harrer et al. [11],

“the key assumption of this approach is that the effect of a comparison, e.g., Reference—Placebo, is exchangeable between trials—no matter if a trial assessed this comparison, or if it is “missing”. In network meta-analyses, ”transitivity” or exchangeability is fulfilled when the effect ${\hat{\theta }}_{i}$ of some comparison is based on a random, independent draw from the overarching distribution of true effects, no matter if this effect size is derived through direct or indirect evidence”.

“The assumption of transitivity can be violated when covariates or other effect modifiers (such as the age group of the studied populations or the treatment intensity) are not evenly distributed across trials assessing, for example, condition R vs. A and A vs. P [17]” (Figure 7). “Transitivity as such cannot be tested statistically, but the risk of violating this assumption can be attenuated by only including studies for which the population, methodology, and target condition are as similar as possible [18]”.

“The statistical manifestation of transitivity is called consistency [19,20]” “Consistency means that the relative effect of comparison (e.g., Reference—Placebo) based on direct evidence does not differ from the one based on indirect evidence [13], i.e.,

$${\hat{\theta }}_{R,P}^{indirect}={\hat{\theta }}_{R,P}^{direct}.”$$

We acknowledge that the assumptions cannot be statistically tested. Therefore, to investigate the impact of deviations from the observed data and to reduce the uncertainty using the margin based on the indirect comparison, several sensitivity analyses are introduced. The focus here is the impact on the preservation factor of the effect size because this is an important part that needs to be aligned with the agencies.

### 4.2. Sensitivity Analysis 1: Direct Comparison Against Similar Drug

While historical trials that allow for direct comparison of reference drug to Placebo are not always available, there may be trial data available that directly compare a drug Anchor, which is similar to Reference in this case, against Placebo (e.g., bioequivalence trial or non-inferiority trials that show a clinically not relevant difference). In this case, we are in the first scenario of Figure 7 and can use this trial to do a direct assessment of the effect size between Anchor and Placebo using the standard approach for margin derivation [3,4].

Given the assumption that the effect size for Reference is indeed similar to the effect size for Anchor, a sensitivity analysis deriving a margin from a direct comparison of Anchor to Placebo can provide assurance on the consistency assumption of the treatment effect between direct and indirect comparison for our indirect margin derivation approach.

### 4.3. Sensitivity Analysis 2: Impact of Variation of Treatment Effect on Reference and Effect Size Reference vs. Anchor

To acknowledge that the true value of the endpoint for Reference can vary across trials, we suggest investigating the impact of the variation of the treatment effect of Reference and of effect size Reference vs. Anchor observed in the Reference vs. Anchor trial on the preservation factor for the equivalence margin. In detail, we suggest the following analyses:

#### 4.3.1. Impact of Variation of the Treatment Effect of Reference

Given the margin we proposed using the anchor-based indirect comparison approach, we can check how much of the effect size we would preserve for varying values of effect in Reference. Hereby, we propose to cover the 95% CI (or level of confidence preferred) for the point estimate of Reference in our trial data, as this gives 95% confidence that we did cover the true value of Reference as well. To be precise, we would propose the following analysis:

1. Fix the effect of Anchor at the point estimate as obtained from the Reference vs. Anchor trial.
2. Vary the effect of Reference across the complete 95% CI for the point estimate of the effect of Reference.
3. For each value of Reference, derive the preservation factor for the margin as derived in Section 2.1 based on point estimates.
4. Graphically display the effect of Reference versus preservation factor.

This will help us understand for which levels of Reference we would still retain enough of the effect size and at which value of Reference this assessment would change. This assessment can further be refined if we also look at the likelihood of obtaining each relevant preservation factor. This could be performed via simulations (for, e.g., 1000 repetitions):

1. Set ${\hat{x}}_{A}$ and ${\hat{x}}_{R}$ to be the same as the treatment point estimates in arms Anchor and Reference with numbers of patients, $n_{A}$ and $n_{R}$, respectively, such that ${\hat{\theta }}_{A,R}^{direct}={\hat{x}}_{A}-{\hat{x}}_{R}$, $E\left[{\hat{x}}_{A}\;\right]=\mu_{A}$ and $E\left[{\hat{x}}_{R}\;\right]=\mu_{R}$.
2. Draw a random value of $\mu_{R}^{*}$ from the posterior distribution of $\mu_{R}$ given the observed data in arm Reference assuming a non-informative prior distribution.
3. Simulate ${\hat{x}}_{R}^{*}$ based on $n_{R}$ patients from the known probability distribution of the point estimate ${\hat{x}}_{R}$ assuming $E\left[{\hat{x}}_{R}\;\right]=\mu_{R}^{*}$.
4. Rederive the preservation factor for the margin according to anchor-based indirect derivation as described above after substituting ${\hat{x}}_{R}$ by ${\hat{x}}_{R}^{*\;}$.
5. Repeat steps (2.) to (4.), e.g., 1000 times.
6. Calculate the probability of retaining at least a target fraction of treatment effect f as the proportion of the 1000 simulation values exceeding or equal to the target fraction f.
7. Graphically display the relationship between the effect size preserved and the probability of retaining at least a certain fraction of the treatment effect.

#### 4.3.2. Impact of Variation of Treatment Difference Reference vs. Anchor

Acknowledging that not only Reference but also Anchor can vary, we also assess the impact of the change in both Reference and <Reference vs. Anchor>, similar to the approach above, but instead of fixing the true value for Anchor, vary the treatment difference of Reference vs. Anchor within a reasonable range and based on the given difference of Reference vs. Anchor assume the value of Anchor.

Varying both assumptions on true treatment effect Reference and effect size Reference vs. Anchor will help us understand the impact of such variations on the preservation factor. In analogy to Section 4.3.1 this assessment can further be refined if we also look at the likelihood to obtain each relevant preservation factor. This could be performed via similar simulations (for, e.g., 1000 repetitions) with the exception that for this sensitivity analysis, the response rates for Anchor and Reference arms in Trial 1 need to be replaced by random draws from their predictive posterior distributions:

1. Set ${\hat{x}}_{A}$ and ${\hat{x}}_{R}$ to be the same as the treatment point estimates in arms Anchor and Reference with numbers of patients, $n_{A}$ and $n_{R}$, respectively, such that ${\hat{\theta }}_{A,R}^{direct}={\hat{x}}_{A}-{\hat{x}}_{R}$, $E\left[{\hat{x}}_{A}\;\right]=\mu_{A}$ and $E\left[{\hat{x}}_{R}\;\right]=\mu_{R}$.
2. Draw a random value of $\mu_{A}^{*}$ and $\mu_{R}^{*}$ from the posterior distribution of $\mu_{A}$ and $\mu_{R}$ given the observed data in the Anchor and Reference arm, respectively, assuming a non-informative prior distribution.
3. Simulate ${\hat{x}}_{A}^{*\;}$ and ${\hat{x}}_{R}^{*}$ based on $n_{A}$ and $n_{R}$ patients from the known probability distribution of the point estimate ${\hat{x}}_{A}$ and ${\hat{x}}_{R}$ assuming $E\left[{\hat{x}}_{A}\;\right]=\mu_{A}^{*}$ and $E\left[{\hat{x}}_{R}\;\right]=\mu_{R}^{*}$, respectively.
4. Rederive the preservation factor for the margin according to anchor-based indirect derivation as described above after substituting ${\hat{x}}_{R}$ by ${\hat{x}}_{R}^{*\;}$.
5. Repeat steps (2.) to (4.), e.g., 1000 times.
6. Calculate the probability of retaining at least a target fraction of treatment effect f as the proportion of the 1000 simulation values exceeding or equal to the target fraction f.
7. Graphically display the relationship between the effect size preserved and the probability of retaining at least a certain fraction of the treatment effect.

### 4.4. Sensitivity Analysis 3: Including Trial Data with Difference in Population Characteristics

When further trial data are available from a trial that studies a slightly different population, these data can be included as a sensitivity analysis to understand the impact of some deviation from the consistency assumption. Such data could be available either for Reference vs. Anchor trials or for Anchor vs. Placebo trials. One would then include such data by first performing a meta-analysis for each arm of the anchor-based approach separately and then having one estimate for each arm following the anchor-based approach as described above.

This will allow for an understanding of the impact of a difference in the relevant population characteristic as available and will further confirm the robustness of data selection for the margin derivation.

## 5. Conclusions

This paper presents a statistical approach to derive the equivalence margin for a biosimilar efficacy trial in special cases where historical data investigating the reference treatment and comparator in the same clinical trial are not available. We implemented a well-known anchor-based indirect comparison approach that, to the best of our knowledge, has not been implemented in this area. This approach enabled us to achieve equivalence margin alignment with the HAs for our pivotal efficacy trial. This is a major achievement because it allowed us to conduct the trial in our preferred combination treatment setting, which allows patients to be treated with a treatment with an optimal safety profile.

## Figures and Tables

**Table 1:** Trial data of Trials 1 and 2.

Study	Treatment	Trial Participants	Responder	Response Rate	95% CI *
Trial 1	Anchor	200	94	0.470	(0.401, 0.539)
	Reference	200	85	0.425	(0.356, 0.494)
Trial 2	Anchor	350	183	0.523	(0.471, 0.575)
	Placebo	350	64	0.183	(0.142, 0.223)

* Calculated as $Rate\pm 1.96*\sqrt{Rate*(1-Rate)/n}.$

**Table 2:** Treatment ratio for included trials and indirect comparison.

Study	Comparison	Ratio of Response Rates	SE(ln(Ratio)) *	95% CI ***
Trial 1	Anchor vs. Reference	1.106	0.111	(0.889, 1.376)
Trial 2	Anchor vs. Placebo	2.859	0.124	(2.242, 3.646)
Indirect comparison	Reference vs. Placebo	2.586	0.167 **	(1.865, 3.585)

* Standard error of response rate ratio (natural logarithmic scale). ** Calculated as $\sqrt{{SE\left(\ln\left({Ratio}_{Trial1}\right)\right)}^{2}+{SE\left(\ln\left({Ratio}_{Trial2}\right)\right)}^{2}}$ with $SE\left(\ln\left(Ratio\right)\right)=\sqrt{1/{Responder}_{Trt1}+1/{Responder}_{Trt2}-1/N_{Trt1}-1/N_{Trt2}}$ and ln() the natural logarithm. *** Calculated as $exp(\ln\left(Ratio\right)\pm 1.96*SE\left(\ln\left(Ratio\right)\right)).$

**Table 3:** Equivalence margin based on the log-transformed lower bound of the 95% CI for indirect comparison.

% Lower Bound Of Maintained Effect Size	Lower 95% CI of Treatment Effect	Equivalence Margin *
40%	1.865	(0.688, 1.454)
50%	1.865	(0.732, 1.366)
60%	1.865	(0.779, 1.283)
70%	1.865	(0.829, 1.206)

* Calculated as $exp\left(\ln\left(lower\;95\%\;CI\;bound\right)\;*\;(100-effect\;size\;retained)/100\right)$ and its inverse.

**Table 4:** Trial data of Trials 2 and 3.

Study	Treatment	N	Responder	Rate	95% CI *
Trial 2	Anchor	350	183	0.523	(0.471, 0.575)
	Placebo	350	64	0.183	(0.142, 0.223)
Trial 3	Anchor	100	53	0.530	(0.432, 0.628)
	Placebo	50	5	0.100	(0.017, 0.183)

* Calculated as $Rate\pm 1.96*\sqrt{Rate*(1-Rate)/n}.$

**Table 5:** Treatment ratio for included trials and indirect comparison.

Study	Comparison	Ratio	SE(lnRatio)	95 % CI **
Trial 1	Anchor vs. Reference	1.106	0.111	(0.889, 1.376)
Trials 2 and 3 combined	Anchor vs. Placebo	3.382	0.275	(1.974, 5.795)
Indirect comparison	Reference vs. Placebo	3.059	0.296 *	(1.711, 5.468)

* Calculated as $\sqrt{{SE\left(\ln\left({Ratio}_{1}\right)\right)}^{2}+{SE\left(\ln\left({Ratio}_{2}\right)\right)}^{2}}$ with $SE\left(\ln\left(Ratio\right)\right)=\sqrt{1/{Responder}_{Trt1}+1/{Responder}_{Trt2}-1/N_{Trt1}-1/N_{Trt2}}$ and ln(), the natural logarithm. ** Calculated as $exp(\ln\left(Ratio\right)\pm 1.96*SE\left(\ln\left(Ratio\right)\right)).$

## Data Availability

The original contributions presented in this study are included in the article/Supplementary Materials. Further inquiries can be directed to the corresponding author.

## Supplementary Materials

The following supporting information can be downloaded at https://www.mdpi.com/article/10.3390/ph18030285/s1: R code, including instructions for the simulations.
