# Supplementary material for: Implementation of the Anchor-Based Indirect Comparison Method for Equivalence Margin Derivation in Biosimilar Development
Source: Pharmaceuticals (Basel). 2025 Feb 20;18(3):285. doi: 10.3390/ph18030285 (PMC11945240; doi:10.3390/ph18030285)

Supplementary document

Implementation of the anchor-based indirect comparison method for equivalence margin derivation in biosimilar development

R code and instructions to perform the simulations

Implementation of simulations

Load packages needed (Note: before using first time it need to be installed)

```
library(netmeta)
```

Read dataset

The following R code prepares a data frame, containing the study information for performing a network meta-analysis.

```
n1 = c(200, 350)
n2 = c(200, 350)
event1 = c(94, 183)
event2 = c(85, 64)
BORR1 = event1/n1
BORR2 = event2/n2
TE= log(BORR1/BORR2)
seTE= sqrt( (1-BORR1)/BORR1/n1 +(1-BORR2)/BORR2/n2 )

MODIF = data.frame(Study=c("Trial 1", "Trial 2"), treat1=c("Anchor",
"Anchor"), treat2=c("Reference", "Placebo"),n1=n1, n2=n2, event1=event1,
event2=event2, BORR1=BORR1, BORR2=BORR2, TE=TE, seTE=seTE )
```

The data contains the following variables:

Study	Study labels
treat1	Label for first treatment
treat2	Label for second treatment

n1	Number of observations in first treatment group.
n2	Number of observations in second treatment group.
event1	Number of events in first treatment group.
event2	Number of events in second treatment group.
BORR1	Response rate in the first treatment group
BORR2	Response rate in the second treatment group
TE	Log risk (response rate) ratio
seTE	Standard error of treatment estimate TE

```
print(MODIF)

    Study treat1    treat2  n1  n2 event1 event2     BORR1     BORR2
TE
1 Trial 1 Anchor Reference 200 200     94     85 0.4700000 0.4250000
0.1006435
2 Trial 2 Anchor   Placebo 350 350    183     64 0.5228571 0.1828571
1.0506031
       seTE
1 0.1113688
2 0.1239968
```

Run a network meta-analysis

```
net <- netmeta(TE, seTE, treat1, treat2, Study, data = MODIF ,  sm = "RR",
random = TRUE)

## Summary results
summary(net)

Original data:

        treat1    treat2     TE   seTE
Trial 1 Anchor Reference 0.1006 0.1114
Trial 2 Anchor   Placebo 1.0506 0.1240

Number of treatment arms (by study):
        narms
Trial 1     2
Trial 2     2

Results (common effects model):

        treat1    treat2     RR           95%-CI    Q leverage
Trial 1 Anchor Reference 1.1059 [0.8890; 1.3756] 0.00     1.00
Trial 2 Anchor   Placebo 2.8594 [2.2425; 3.6460] 0.00     1.00

Results (random effects model):
```

```
        treat1    treat2     RR           95%-CI
Trial 1 Anchor Reference 1.1059 [0.8890; 1.3756]
Trial 2 Anchor   Placebo 2.8594 [2.2425; 3.6460]

Number of studies: k = 2
Number of pairwise comparisons: m = 2
Number of treatments: n = 3
Number of designs: d = 2

Common effects model

Treatment estimate (sm = 'RR', comparison: other treatments vs 'Anchor'):
              RR           95%-CI     z  p-value
Anchor         .                .     .        .
Placebo   0.3497 [0.2743; 0.4459] -8.47 < 0.0001
Reference 0.9043 [0.7269; 1.1248] -0.90   0.3662

Random effects model

Treatment estimate (sm = 'RR', comparison: other treatments vs 'Anchor'):
              RR           95%-CI     z  p-value
Anchor         .                .     .        .
Placebo   0.3497 [0.2743; 0.4459] -8.47 < 0.0001
Reference 0.9043 [0.7269; 1.1248] -0.90   0.3662

Quantifying heterogeneity / inconsistency:
tau^2 = NA; tau = NA

Tests of heterogeneity (within designs) and inconsistency (between designs):
                Q d.f.  p-value
Total           0    0       --
Within designs  0    0       --
Between designs 0    0       --

## forest plot
plot(net)
```

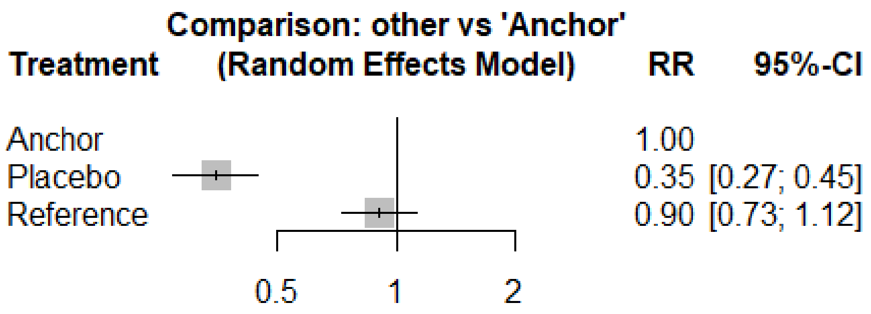

Sensitivity analysis: Impact of variation of the treatment effect of Reference

Implementation of steps (1) to (7) described in Subsection 4.3.1.

Implementation of steps (1) to (4).

The R function, *sanafa()* reruns a network meta-analysis after substituting the number of events in the reference arm, event2 by a random draw from its posterior predictive distribution, then it derives a preservation factor corresponding to the upper margin of 1.283. The function *sanafa* returns the following values, response rate (q) in the Reference arm, Risk ratio (RR) of Reference vs Placebo, lower limit of 95% CI of RR (RR.lower), upper limit of 95% CI of RR (RR.upper) and the fraction of treatment to retain (F) corresponding to a margin 1.283, i.e., $$log(RR.lower)*(1-F) = log(1.283)$$

```
SADSET=MODIF
sanafa<-function(){
  n12 <- MODIF[1, "n2"]
  Q <- rbeta(1, MODIF[1, "event2"], n12-MODIF[1, "event2"])
  y <- rbinom(1,n12, Q)
  q <- y/n12
  n11 <- MODIF[1, "n1"]
  p <-  MODIF[1, "BORR1"]

  SADSET[1, "event2"] <- y
  SADSET[1, "BORR2"] <- q
  SADSET[1, "TE"] <- log( p/ q)
  SADSET[1, "seTE"] <- sqrt( (1-p)/p/n11 +  (1-q)/q/n12 )

  net <- netmeta(TE, seTE, treat1, treat2, Study, data = SADSET,  sm = "RR",
random = TRUE)

   Fr <- 1-log(1.283) /net$lower.indirect.random[3,2]
   RR= exp(net$TE.indirect.random[3,2])
   RR.lower= exp(net$lower.indirect.random[3,2])
   RR.upper = exp(net$upper.indirect.random[3,2])

  c(q=q, RR= RR, RR.lower= RR.lower, RR.upper = RR.upper, F=Fr)
}
```

Implementation of step (5).

The next R code runs the function *sanafa* 1000 times using the R function *replicate* to obtain a 1000 by 6 matrix including values of the variables, q, RR, RR.lower, RR.upper and F (see above for the description of the variables)

```
# Set the seed for reproducibility
set.seed(12122024)
mn <- replicate(1000, sanafa())
mn <- t(mn)
```

Implementation of steps (6) and (7).

The following chunk of code calculates the probability that the assumed margin 1.283 ensures at least preservation of a fraction, x of the effect size of reference arm vs Placebo, as a proportion of the replicates for which $$F > x$$, for a range of x values in (0.05, 0.95). Then it plots the resulted probability against a range of preservation fractions.

```
z <- mn[,"F"]

# Probability to preserve at least 50% (60%) effect size
# using a margin (0.779, 1.283)
mean(z>0.5)

[1] 0.82

mean(z>0.6)

[1] 0.506

a=seq(0.05,0.95, 0.01 )
b= sapply(a, function(x) mean(z>x))

plot(a,b, type="l",xlab="F: Target fraction of treatment to retain",
ylab="Probability", main= "Figure 3: Probability of retaining at least a
target fraction F", col=3, lwd=3, cex.main=0.9, cex.lab=0.9, cex=0.9)
abline( v=0.5, h=mean(z>0.5), lty=2, col=8)
abline( v=0.6, h=mean(z>0.6), lty=2, col=8)
```

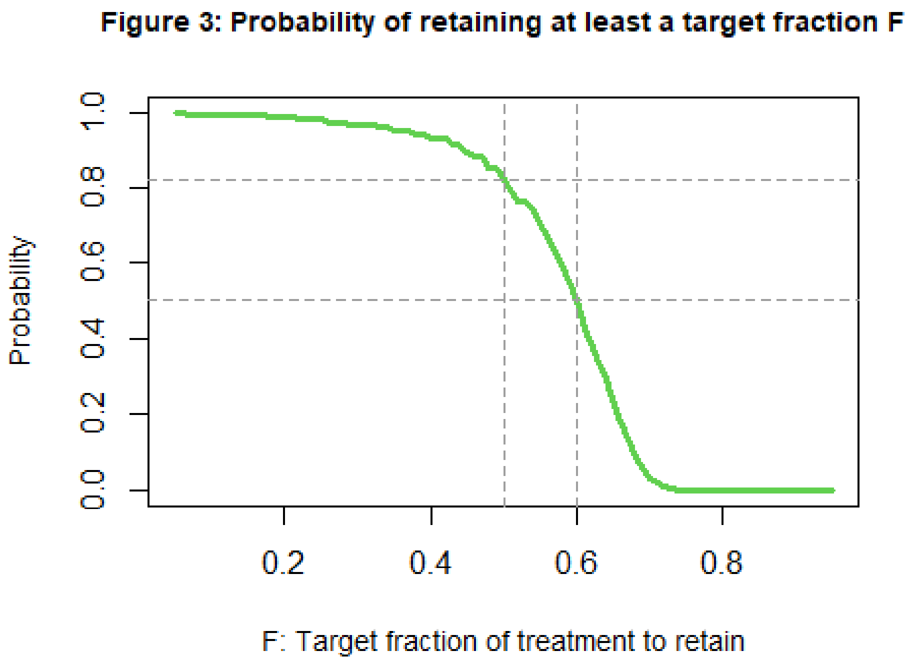

Sensitivity analysis: Impact of variation of treatment difference Reference vs Anchor

Implementation of steps (1) to (7) described in Subsection 4.3.2.

Implementation of steps (1) to (4).

The R function, *sanafb()* reruns a network meta-analysis after substituting the number of events, event1 and event2 in Anchor and the reference arm by a random draws from their posterior predictive distributions, respectively. Then it derives a preservation factor corresponding to the upper margin of 1.283. The function *sanafb* returns the following values, response rate (q1) in the Anchor arm for trial 1, response rate (q2) in the Reference arm for trial 1, Risk ratio (RR) of Reference vs Placebo, lower limit of 95% CI of RR (RR.lower), upper limit of 95% CI of RR (RR.upper) and the fraction of treatment to retain (F) corresponding to a margin 1.283, i.e., $$log(RR.lower)*(1-F) = log(1.283)$$.

```
SADSET=MODIF
sanafb<-function(){
  n11=MODIF[1, "n1"]
  n12=MODIF[1, "n2"]
  Q1=rbeta(1, MODIF[1, "event1"], n11 - MODIF[1, "event1"])
  Q2=rbeta(1, MODIF[1, "event2"],  n12 - MODIF[1, "event2"])
  y1=rbinom(1,n11, Q1)
  y2=rbinom(1,n12, Q2)
  q1=y1/n11
  q2=y2/n12
  SADSET[1, "event1"]= y1
  SADSET[1, "BORR1"]= q1
  SADSET[1, "event2"]= y2
  SADSET[1, "BORR2"]= q2
  SADSET[1, "TE"]= log( q1/ q2)
  SADSET[1, "seTE"]= sqrt( (1-q1)/q1/n11 +  (1-q2)/q2/n12 )

  net <- netmeta(TE, seTE, treat1, treat2, Study, data = SADSET,  sm = "RR", 
random = TRUE)
  Fr= 1-log(1.283) /net$lower.indirect.random[3,2]
  RR= exp(net$TE.indirect.random[3,2])
  RR.lower= exp(net$lower.indirect.random[3,2])
  RR.upper = exp(net$upper.indirect.random[3,2])

  c(q1=q1, q2=q2, RR= RR, RR.lower= RR.lower, RR.upper = RR.upper, F=Fr)
}
```

Implementation of step (5).

The next R code chunk runs the function *sanafb* 1000 times using the R function *replicate* to obtain a 1000 by 6 matrix including values of the variables, q1, q2, RR, RR.lower, RR.upper and F (see above for the description of the variables)

```
# Set the seed for reproducibility
set.seed(12122024)
mn <- replicate(1000, sanafb())
mn <- t(mn)
```

Implementation of steps (6) and (7).

The following chunk of code calculates the probability that the assumed margin 1.283 ensures at least preservation of a fraction, x of the effect size of reference arm vs Placebo, as a proportion of the replicates for which $$F > x$$, for a range of x values in (0.05, 0.95). Then it plots the resulted probability against a range of preservation fractions.

```
zz= mn[,"F"]

# Probability to preserve at least 50% (60%) effect size
# using a margin (0.779, 1.283)
mean(zz>0.5)

[1] 0.767

mean(zz>0.6)

[1] 0.502

a=seq(0.05,0.95, 0.01 )
b= sapply(a, function(x) mean(zz>x))

plot(a,b, type="l",xlab="F: Target fraction of treatment to retain",
ylab="Probability", main= "Figure 5: Probability of retaining at least a
target fraction F", cex.main=0.9, cex.lab=0.9, cex=0.9)
abline( v=c(0.5, 0.6), h=c(mean(zz>0.5), mean(zz>0.6)), lty=2, col=8)
```

Figure 5: Probability of retaining at least a target fraction F

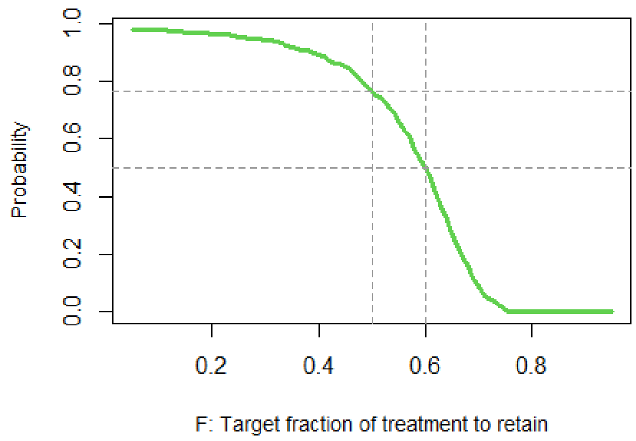

Supplement: Supplementary file 1 [file pharmaceuticals-18-00285-s001.zip › pharmaceuticals-3409146-supplementary.pdf]
